# Dual Chain-Mediation of GenAI Chatbots on Loneliness: Perceived Misinformation Exposure and User Trust via Negative Emotions

**DOI:** 10.3389/ijph.2026.1609017

**Published:** 2026-03-17

**Authors:** Yangyang Wang, Chang Xu

**Affiliations:** 1 School of Communication, Soochow University, Suzhou, China; 2 School of Journalism, Fudan University, Shanghai, China; 3 School of Literature and History (School of Journalism and Communication), Qiqihar University, Qiqihar, China

**Keywords:** GenAI chatbots, loneliness, negative emotions, perceived misinformation exposure, user trust

## Abstract

**Objectives:**

Mounting concerns over “AI psychosis” highlight the need to examine psychological risks of GenAI chatbots. Loneliness is a critical outcome of prolonged AI use, often mediated by distorted emotional and cognitive processes. This study tests how GenAI chatbots use is associated with negative emotions, which in turn heighten loneliness through two concurrent mechanisms.

**Methods:**

We surveyed 516 adults online and applied PLS-SEM with bootstrapped indirect effects and comparative pathway analysis.

**Results:**

GenAI chatbots use significantly predicted perceived misinformation exposure (β = 0.318, 95% CI [0.231, 0.409]) and user trust (β = 0.383, 95% CI [0.294, 0.473]). Both pathways increased loneliness via negative emotions, with the information-quality pathway (β = 0.062, 95% CI [0.036, 0.099]) stronger than the emotional-trust pathway (β = 0.023, 95% CI [0.009, 0.040]); overall chain effects did not differ.

**Conclusion:**

GenAI chatbots use contributes to loneliness through dual cognitive and emotional mediations. Given emerging AI psychosis risks, interventions should strengthen misinformation recognition and address trust-related vulnerabilities.

## Introduction

Generative Artificial Intelligence (GenAI) has seen rapid adoption in recent years, particularly conversational AI chatbots such as ChatGPT, DeepSeek, and Claude, which have increasingly integrated into learning, work, and daily communication [1, 2]. The convenience of interaction, information generation, and emotional companionship it offers have driven its rapid adoption among the general public [3–5]. However, the explosive application of this technology has also raised concerns about the emerging phenomenon of “AI psychosis (ChatGPT psychosis)”. AI psychosis refers to the phenomenon where some individuals, after prolonged interaction with AI chatbots, experience hallucinations or delusions, emotional dependence, or a loss of reality. In the field of public health, scholars have long been concerned about the connection between virtual social interaction and mental health [6–8], but empirical research on how GenAI chatbots influence loneliness through specific psychological and social mechanisms remains limited [9, 10]. Existing studies primarily focus on observational and conceptual analyses, lacking systematic theoretical frameworks and mechanism testing.

In the field of academic research, public health studies have long recognized loneliness as a major global health concern [11], closely linked to cardiovascular disease, depression, and cognitive decline [12–15]. However, the mechanisms triggering loneliness in contemporary society are undergoing transformation. In the digital environment, artificial intelligence tools not only alter human-machine interaction but also reshape individuals’ perceptions of information, trust, and social existence [16–18]. Specifically, in the use of GenAI chatbots, users on one hand gain information and emotional support through real-time interaction [16, 19], but on the other hand, they may face information overload, perceived exposure to misinformation, and the complex process of building trust in AI [20]. If the perceived misinformation exposure is high, it may lead to cognitive imbalance in users, thereby intensifying negative emotional experiences [21]. If trust relationships are imbalanced, it is associated with emotional frustration or even social isolation [22, 23]. Therefore, there is an urgent need to construct a systematic theoretical model to reveal the chain-reaction mechanisms between GenAI chatbots use, perceived misinformation exposure, user trust, emotional responses, and loneliness.

Based on the above background, this study attempts to explore the relationship between GenAI chatbots use and loneliness from an interdisciplinary perspective combining public health and social psychology. It integrates the Stimulus-Organism-Response (S-O-R) theory [24], the Social Presence Theory [25], and the technology acceptance and use theory (TAM/UTAUT) [26, 27], to construct an integrated framework exploring how perceived misinformation exposure and user trust are linked to individual emotional states in the use of GenAI chatbots, thereby affecting loneliness.

### Theoretical Analysis and Hypothesis Development

To explain the psychological mechanisms associated with GenAI chatbots use, this study integrates three perspectives: the Stimuli–Organism–Response (S-O-R) model, social presence theory, and the technology acceptance tradition (TAM/UTAUT). The S-O-R model proposes that external stimuli (S) are associated with individuals’ cognitive and emotional processing (O), which in turn relates to subsequent psychological responses (R) [24]. In this study, GenAI chatbots use is conceptualized as a stimulus, while cognitive processing is reflected in users’ evaluation of information quality (operationalized as perceived misinformation exposure) and user trust, and emotional processing is reflected in negative emotions; loneliness is considered a psychological response.

First, regarding the information-quality pathway, GenAI chatbots’ responses can be fluent and novel but may also contain fictional or uncertain content [28]. Health communication research suggests that the ability to discern information is especially important under information overload and misinformation [29, 30]. When users repeatedly encounter ambiguous or contradictory content during interactions, they may experience cognitive dissonance and emotional distress, such as anxiety, frustration, and helplessness [29, 31, 32]. Such negative emotions are associated with reduced willingness to engage in social interaction and may be linked to greater reliance on machine-mediated interaction, which can coincide with heightened loneliness [33–35]. Accordingly, we propose that GenAI chatbots use is positively associated with loneliness through an indirect pathway involving perceived misinformation exposure and negative emotions.

Second, regarding the emotional-trust pathway, users may develop a sense of social presence during interactions with GenAI chatbots based on perceived performance and anthropomorphic cues [25, 36]. Social presence theory suggests that when an interactive entity is perceived as having social attributes, users may develop anthropomorphic trust and attachment [25, 37]. However, trust may operate as a double-edged mechanism [38]. While moderate trust can enhance interaction experiences, excessive trust may coincide with neglecting a system’s limitations, potentially intensifying negative experiences when errors are encountered [39, 40]. Drawing on emotional appraisal perspectives, such trust-related emotional reactions may be associated with increased loneliness [31, 41]. Accordingly, we propose that GenAI chatbots use is positively associated with loneliness through an indirect pathway involving user trust and negative emotions.

Further, TAM and UTAUT emphasize that technology adoption is shaped not only by perceived usefulness and ease of use but also by perceived trust, perceived risk, and social influence [26, 27]. In the context of GenAI, user trust and information discernment are intertwined with cognitive processing and emotional responses, forming a complex psychological pattern [42]. Integrating the above perspectives, we propose a dual-chain mediation framework comprising (a) an “information-quality path” (GenAI chatbots use→perceived misinformation exposure→negative emotions→ loneliness) and (b) an “emotional-trust path” (GenAI chatbots use→user trust→negative emotions →loneliness). Based on this framework, we state the following hypotheses in terms of associations and indirect effects:

H1 GenAI chatbots use is positively associated with perceived misinformation exposure.

H2 GenAI chatbots use is positively associated with user trust.

H3 Perceived misinformation exposure is positively associated with negative emotions, which are positively associated with loneliness (indirect association).

H4 User trust is positively associated with negative emotions, which are positively associated with loneliness (indirect association).

H5 GenAI chatbots use is positively associated with loneliness through the indirect pathway of perceived misinformation exposure and negative emotions.

H6 GenAI chatbots use is positively associated with loneliness through the indirect pathway of user trust and negative emotions.

**FIGURE 1:**
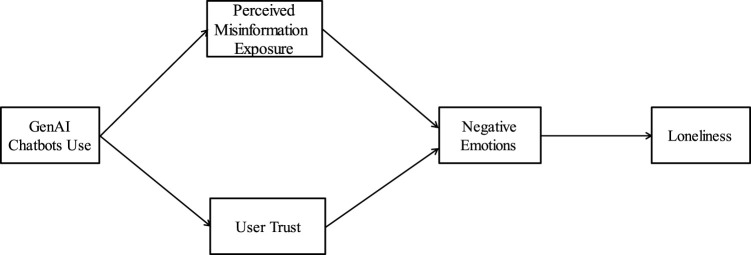
Theoretical framework (China, 2026).

The theoretical framework formed through the above theoretical integration and hypothesis derivation is shown in Figure 1.

## Methods

### Data Collection

This study employed a cross-sectional design and conducted an online survey via the WenJuanXing platform (www.wjx.cn) in December 2024. Respondents were recruited as a non-probability online convenience sample and screened to include active users who frequently interacted with GenAI chatbots (e.g., ChatGPT, DeepSeek). A total of 550 questionnaires were collected. After data quality screening, we excluded 4 responses that failed attention-check items and 30 responses that showed abnormal response patterns (implausibly short completion time), resulting in 516 valid questionnaires. The demographic characteristics of the sample (e.g., age, gender, education, income) are reported in Table 1. Given the online, non-probability nature of the sample, the findings should be interpreted as most relevant to active GenAI users in China who participated in this survey rather than as representative of the general population.

**TABLE 1:** Respondent’s characteristics (N = 516) (China, 2026).

Variable	Type of statistics	Statistics
Gender		
Female	n (%)	193 (37.40%)
Male	n (%)	323 (62.60%)
Age	Mean (SD)	31.54 (5.75)
Marriage status		
Unmarried	n (%)	212 (41.09%)
Others	n (%)	304 (58.91%)
Educational attainment	Mean (SD)	15.76 (2.49)
Length of employment	Mean (SD)	7.28 (5.09)
Average monthly income		
Under ¥1,000	n (%)	2 (0.39%)
¥1,001- ¥3,000	n (%)	17 (3.29%)
¥3,001- ¥5,000	n (%)	139 (26.94%)
¥5,001- ¥10,000	n (%)	224 (43.41%)
¥1,001- ¥15,000	n (%)	79 (15.31%)
¥15,001- ¥20,000	n (%)	37 (7.17%)
¥20,001 or more	n (%)	18 (3.49%)

### Measures

All latent variables in this study were measured using established scales developed in existing international literature, and underwent a standard “translation-back translation” procedure to ensure conceptual equivalence and semantic clarity in the Chinese cultural context. All variables were scored using a five-point Likert scale (1 = “Strongly Disagree,” 5 = “Strongly Agree”), except for GenAI chatbots use. Specifically, these include:

#### Loneliness (LL)

Loneliness was measured using the 6-item loneliness scale (ULS-6) recently revised by Xiao and Du for the Chinese context [43].

#### GenAI Chatbots Use (GCU)

GenAI chatbots use is an observable variable, measured using two items to assess the intensity of respondents’ use of GenAI chatbots (How often have you used domestic GenAI chatbots such as DeepSeek, DouBao, Kimi, etc. in the past 12 months; How often have you used foreign GenAI chatbots such as ChatGPT, Claude, Gemini, etc. in the past 12 months?) captured using a 7-point Likert scale (Never used; Used less than 1 week; Used 1 week to 1 month; Used 1 month to 3 months; Used 3 months–6 months; Used 6 months to 1 year; Used more than 1 year).

#### Perceived Misinformation Exposure (PME)

PME was measured using five items adapted from Xiao et al. [44]. The items capture respondents’ perceived frequency of encountering misleading, fabricated, or difficult-to-verify GenAI outputs. Higher scores indicate higher perceived exposure to misleading GenAI-generated information.

#### User Trust (UT)

User trust is measured using scales adapted from Bedi and Vashisth [45] and Shin [42]. In the measurement model assessment, the UT scale showed acceptable composite reliability (CR = 0.811) and convergent validity (AVE = 0.589), with item loadings ranging from 0.714 to 0.799. Although Cronbach’s alpha was 0.654, we interpret UT as adequate for exploratory PLS-SEM.

#### Negative Emotions (NE)

Negative emotions were measured using the scale developed by Rodway et al. [46] and the measurement method by Acosta-Enriquez et al. [47].

### Common Method Bias

As all data were collected at a single time point using self-report questionnaires, common method bias (CMB) cannot be fully ruled out. We applied Harman’s single-factor test as a preliminary diagnostic; the first unrotated factor accounted for 25.86% of the total variance, below the commonly used 50% threshold [48]. Recognizing that Harman’s test alone is limited, we additionally assessed CMB using a PLS-SEM–compatible diagnostic by examining full collinearity VIFs. All construct VIF values were below 3.1 (see Table 3, maximum VIF = 3.008), suggesting that CMB is unlikely to be a serious threat to the results. In the survey design, we also included attention-check items and excluded responses that failed these checks or exhibited abnormal response patterns.

### Statistical Analysis

Partial Least Squares Structural Equation Modeling (PLS-SEM) was employed for model and hypothesis testing with SmartPLS 4 [49]. PLS-SEM was selected primarily because it does not require data to strictly adhere to a normal distribution and is more suitable for predictive analysis and handling complex models [50, 51]. The data in this study did not strictly follow a normal distribution and were part of an exploratory study. The empirical analysis followed a two-stage testing process for the measurement model and structural model: first, Cronbach’s Alpha, CR, AVE, and HTMT and Fornell-Larcker indicators were used to test the reliability, convergent validity, and discriminant validity of the scales; subsequently, Bootstrap resampling (5,000 times) was used to test the path coefficients and their significance in the structural model. In addition, we estimated a competing model that added the direct path GCU→LL and compared the explained variance in loneliness between models (ΔR^2^), using the same bootstrapping settings.

## Results

### Sample Characteristics

This study analyzed the demographic characteristics of 516 valid samples and found that the sample was predominantly young, highly educated, and of moderate income (Table 1). Specifically, the average age of respondents was 31.54 years (SD = 5.75), with 62.6% being male. The majority of respondents were married or in other marital statuses (58.91%). In terms of educational attainment, the average years of education was 15.76 years (SD = 2.49), equivalent to a bachelor’s degree or higher. The average years of work experience was 7.28 years (SD = 5.09), indicating that the respondents had substantial work experience. Income distribution shows that 70.35% of respondents have monthly incomes concentrated in the ¥3,001–¥10,000 range, with the ¥5,001–¥10,000 group accounting for the highest proportion (43.41%), while those with monthly incomes exceeding ¥10,000 collectively account for 25.97%. This demographic profile suggests that our respondents largely reflect younger, highly educated, and moderate-income active GenAI users recruited online. While this composition is consistent with the characteristics of early adopters of digital technologies, it should not be interpreted as representative of the general Chinese population. Accordingly, the findings should be generalized primarily to active GenAI users in China who are similar to those recruited via WenJuanXing.

### Measurement Model Validation

PLS-SEM was used to test the reliability and validity of the measurement model. As shown in Table 2, all measurement indicators for the constructs demonstrated good psychometric properties. First, the factor loadings (Loadings) for each measurement item were all above the recommended standard of 0.70 (ranging from 0.714 to 0.892), indicating that the scale has good item reliability. Second, the composite reliability (CR) values for all constructs ranged from 0.811 to 0.936, and Cronbach’s Alpha ranged from 0.654 to 0.919, both exceeding the threshold of 0.65 (according to Nunnally’s classical standard, Cronbach’s Alpha greater than 0.60 is acceptable in exploratory research) [52], indicating excellent internal consistency reliability for each scale. The average variance extracted (AVE) values all exceeded the 0.50 criterion (ranging from 0.811 to 0.936), confirming the measurement model’s good convergent validity. Additionally, the variance inflation factor (VIF) values for all variables are well below the strict critical value of 3.1, indicating that multicollinearity issues do not have a substantial impact on the model estimation results.

**TABLE 2:** Reliability and validity of the measurement model (China, 2026).

Variable	Loadings factor	AVE	CR	Cronbach’s alpha	VIF
Loneliness					
LL1: In the last week, have you often felt like you lacked a partner?	0.831	0.710	0.936	0.919	2.266
LL2: In the last week, have you often felt like you have no one to rely on?	0.847	​	​	​	2.767
LL3: In the last week, have you often felt left out?	0.854	​	​	​	2.786
LL4: Have you felt alienated from others in the last week?	0.824	​	​	​	2.461
LL5: In the last week, have you often felt unhappy because you were alone?	0.809	​	​	​	2.388
LL6: In the last week, have you often felt surrounded by people but no one cares about you?	0.888	​	​	​	3.008
Perceived misinformation exposure					
MI1: When using GenAI chatbots such as Chatgpt, DeepSeek, etc., I have often encountered fabricated and misleading information that feels grossly distorted or unfounded	0.835	0.642	0.900	0.861	2.005
MI2: When using GenAI chatbots such as Chatgpt, DeepSeek, etc., I have often encountered information that has been altered and plausibly reassembled from sources elsewhere	0.775	​	​	​	1.705
MI3: When using GenAI chatbots such as Chatgpt, DeepSeek, etc., I often encounter misleading opinions or information generated in the name of a professional authority or person	0.797	​	​	​	1.858
MI4: When using GenAI chatbots such as Chatgpt, DeepSeek, etc., I often encounter responses that appear to be truthful and persuasive, making it easy to mistake them for correct facts	0.803	​	​	​	1.906
MI5: When using GenAI chatbots such as Chatgpt, DeepSeek, etc., I have often encountered responses to information that cater to my cultural habits, making it difficult to detect the truthfulness of the information	0.795	​	​	​	1.849
User trust					
UT1: I trust the information generated by GenAI chatbots	0.799	0.589	0.811	0.654	1.319
UT2: The information obtained through GenAI chatbots is trustworthy	0.788	​	​	​	1.256
UT3: I Trust the information generated by GenAI chatbots to be reliable	0.714	​	​	​	1.261
Negative emotions					
NE1: I do not like the idea of using GenAI chatbots to replace certain human skills such as reasoning, information searching, analyzing, and writing	0.823	0.738	0.894	0.822	1.681
NE2: I am concerned that frequent use of GenAI chatbots will limit my ability to think and solve problems independently	0.892	​	​	​	2.142
NE3: I am concerned that excessive use of GenAI chatbots will reduce my interest in researching and reading a variety of information sources	0.861	​	​	​	1.902

To further test discriminant validity, this study employed both the HTMT and Fornell-Larcker criteria. As shown in Table 3, all HTMT values were below the conservative threshold of 0.85 (ranging from 0.131 to 0.631), further confirming the good discriminant validity among the constructs. Additionally, the square root of the AVE (bolded diagonal values) for each construct was greater than the correlation coefficient between that construct and other constructs, meeting the requirements of the Fornell-Larcker criterion.

**TABLE 3:** Discriminant validity: Heterotrait-Monotrait Ratio of Correlations (HTMT) and Fornell-Larcker (China, 2026).

​	LL	GCU	PME	UT	NE
**HTMT**					
LL					
GCU	0.131	​	​	​	​
PME	0.164	0.458	​	​	​
IT	0.240	0.631	0.294	​	​
NE	0.205	0.220	0.428	0.278	​
**Fornell-larcker**					
LL	**0.844**	​	​	​	​
GCU	0.036	**0.828**	​	​	​
PME	0.145	0.322	**0.800**	​	​
UT	−0.190	0.383	0.231	**0.767**	​
NE	0.188	0.148	0.361	0.199	0.859

LL, Loneliness; GCU, GenAI Chatbots Use; PME, Perceived Misinformation Exposure; UT, Information Trust; NE, Negative Emotions.

### Hypothesis Testing

**FIGURE 2:**
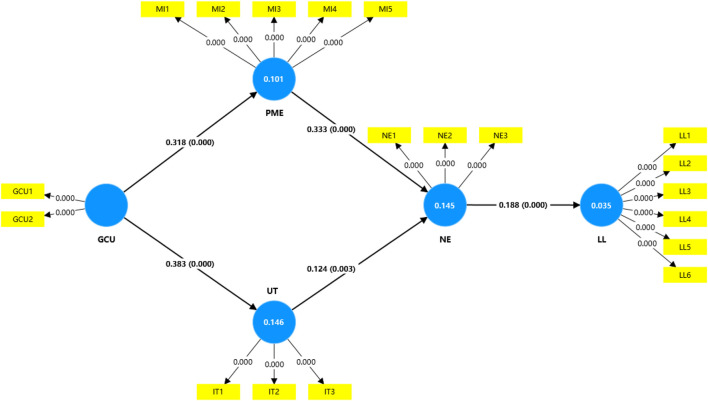
Output bootstrapping of Partial Least Squares Structural Equation Modeling (PLS-SEM) results (generated using SmartPLS 4; [49]; China, 2026).

PLS-SEM-based bootstrap analysis (5,000 times) supported all research hypotheses in this study, as shown in Figure 2 and Table 4. As shown in Table 4, GenAI chatbots use (GCU) has a significant positive predictive effect on perceived misinformation exposure (PME) (β = 0.318, t = 7.094, p < 0.001, 95% CI [0.231, 0.409]), supporting hypothesis H1. Additionally, GCU exhibits a significant positive predictive effect on user trust (UT) (β = 0.383, t = 8.487, p < 0.001, 95% CI [0.294, 0.473]), validating hypothesis H2.

**TABLE 4:** Results of hypothesis testing about total effects, direct effects and indirect effects (China, 2026).

Structural path	Path coefficient	T-test	P values	Confidence interval (95%)	Results
Total effects					
GCU -> LL	0.029	4.087	0.000	[0.018–0.045]	Significant
Direct effects					
GCU -> PME	0.318	7.094	0.000	[0.231–0.409]	Significant
GCU -> UT	0.383	8.487	0.000	[0.294–0.473]	Significant
PME -> NE	0.333	6.159	0.000	[0.227–0.440]	Significant
UT -> NE	0.124	2.955	0.003	[0.044–0.210]	Significant
NE -> LL	0.188	5.654	0.000	[0.129–0.258]	Significant
Indirect effects					
PME -> NE -> LL (Ind1)	0.062	3.829	0.000	[0.036–0.099]	Significant
UT -> NE -> LL (Ind2)	0.023	3.037	0.002	[0.009–0.040]	Significant
Path difference (Δ = ind1 - ind2)	0.039	9.128	0.001	[0.032–0.045]	Significant
GCU -> PME -> NE -> LL (Ind3)	0.020	3.139	0.002	[0.010–0.035]	Significant
GCU -> UT -> NE -> LL (Ind4)	0.009	2.714	0.007	[0.003–0.016]	Significant
Path difference (Δ = ind3 - ind4)	0.012	1.618	0.054	[-0.001–0.027]	Not significant

LL, Loneliness; GCU, GenAI Chatbots Use; PME, Perceived Misinformation Exposure; UT, Information Trust; NE, Negative Emotions. Parameter estimates were based on bootstrapping with 5,000 resamples; 95% percentile bootstrap confidence intervals are reported.

In terms of the emotional transmission mechanism, perceived misinformation exposure (PME) significantly and positively predicted negative emotions (NE) (β = 0.333, t = 6.159, p < 0.001, 95% CI [0.227, 0.440]), supporting hypothesis H3. User trust (UT) also had a significant positive effect on negative emotions (NE) (β = 0.124, t = 2.955, p = 0.003, 95% CI [0.044, 0.210]), confirming hypothesis H4. Negative emotions (NE) significantly and positively predict loneliness (LL) (β = 0.188, t = 5.654, p < 0.001, 95% CI [0.129, 0.258]).

Results of the analysis of indirect effects indicate that the simple mediating effects of negative emotions are significant. Specifically, the indirect effect value for path Ind1 is 0.062 (95% CI [0.036, 0.099]), and the indirect effect value for path Ind2 is 0.023 (95% CI [0.009, 0.040]). Additionally, both chain-mediated paths were significant, with the indirect effect value of path Ind3 being 0.020 (95% CI [0.010, 0.035]), supporting Hypothesis H5; and the indirect effect value of path Ind4 being 0.009 (95% CI [0.003, 0.016]), supporting Hypothesis H6. Further testing of path differences revealed that the effect strength of path Ind1 was significantly greater than that of path Ind2 (Δ = 0.039, p = 0.001, 95% CI [0.032, 0.045]), but the effect difference between the two chained mediating paths, Path Ind3 and Path Ind4, did not reach statistical significance (Δ = 0.012, p = 0.054, 95% CI [-0.001, 0.027]).

In summary, all six hypotheses proposed in this study were statistically validated. The total effect of the model shows that GCU has a significant positive effect on LL (β = 0.029, p < 0.001, 95% CI [0.018, 0.045]), indicating that GenAI chatbots exert a statistically significant effect on users’ loneliness through parallel multiple mediating mechanisms involving perceived misinformation exposure, user trust, and negative emotions.

Besides, in a competing model that additionally included a direct path from GCU to loneliness, the direct effect was not statistically significant (β = 0.006, t = 0.126, p = 0.900, 95% CI [−0.090, 0.098]). The explained variance in loneliness did not increase after adding this direct path (R^2^ remained 0.035; ΔR^2^ = 0), while the hypothesized serial indirect effects remained statistically supported.

## Discussion

In this study, we aim to reveal how GenAI chatbots use indirectly is associated with users’ emotional states through information quality and affective trust mechanisms, ultimately linking to loneliness—which is a critical public health issue. Empirical results confirm all research hypotheses, indicating that GenAI chatbots use transcends mere technological application. Instead, it profoundly shapes users’ psychological experiences through intricate cognitive and emotional mechanisms [41]. These findings not only expand our understanding of the relationship between GenAI chatbots use and mental health but also provide new evidence for public health research on AI psychosis risks.

We first found that GenAI chatbots use significantly and positively predicted users’ perceived misinformation exposure (H1), consistent with existing literature on information processing and cognitive load [53, 54]. Unlike traditional social media, GenAI chatbots deliver interactive and generative content. Users become more attuned to potential information biases during conversations with the system, thereby stimulating stronger critical thinking [55, 56]. This finding suggests that GenAI chatbots is not linked to perceived misinformation exposure; rather, their interactive nature may enhance users’ discernment capabilities to some extent. However, this recognition carries an emotional cost: when users perceive potential misinformation, it can generate cognitive uncertainty and negative emotions (H3), thereby increasing loneliness. That is, during interactions with GenAI, perceived misinformation exposure links cognitive uncertainty, prompting users to allocate more cognitive resources to critical processing [57]. However, excessive critical processing, while strengthening cognitive control, may deplete emotional regulation resources, thereby inducing negative emotions [31, 58, 59]. Simultaneously, this overconsumption of cognitive resources weakens emotional regulation capacity, leading to heightened loneliness [60].

Secondly, GenAI chatbots use positively predicted users’ trust in AI systems (H2). This finding aligns with predictions from the Technology Acceptance Model (TAM) and the Unified Theory of Acceptance and Use of Technology (UTAUT), which suggest that interactive and anthropomorphic designs enhance users’ perceived credibility and reliance [26, 27, 61]. However, this study further reveals that such trust is not an entirely positive psychological resource. On the contrary, it may introduce new psychological risks by reinforcing users’ dependence on GenAI chatbots [38]. Results indicate that user trust indirectly contributes to increased negative emotions, ultimately leading to loneliness (H4) [41]. This pathway suggests that while user trust in GenAI chatbots fosters user-system relationships, users may experience emotional gaps within this dependency when deprived of authentic social interaction substitutes, thereby intensifying loneliness [62]. This phenomenon aligns with the “digital substitution hypothesis”, which posits that enhanced virtual interactions do not equate to fulfilling genuine social connections but may instead mask or exacerbate the core nature of loneliness [35, 63].

Further chain mediation analysis confirmed that both pathways of perceived misinformation exposure → negative emotions → loneliness (H5) and user trust → negative emotions → loneliness (H6) were established. This finding reveals two parallel mechanisms: First, the information quality pathway emphasizes negative emotions triggered when users recognize uncertainty and potential errors during cognitive processing; Second, the trust pathway highlights the loneliness experienced when users emotionally depend on GenAI chatbots yet lack authentic social support. Together, these pathways constitute the core mechanism through which GenAI chatbots use impacts mental health. This finding deepens the application of the SOR model, revealing how external technological stimuli (GenAI chatbots use) is linked to users’ mental health outcomes via dual cognitive and emotional pathways [24].

However, it is noteworthy that the two mechanisms show different effect patterns depending on the level of comparison. When focusing on the downstream indirect segment linking the mediators to loneliness, the indirect effect via perceived misinformation exposure → negative emotions → loneliness (Ind1; corresponding to H3) was larger than that via user trust → negative emotions → loneliness (Ind2; corresponding to H4). This pattern suggests that, in the context of GenAI chatbots use, information-quality–related cognitive processing may be more strongly associated with loneliness through negative emotions than trust-related processing. This interpretation is consistent with the “negativity bias” in affective psychology, whereby individuals tend to be more sensitive to negative cues than positive ones [64, 65].

Importantly, when comparing the two full serial indirect effects from GenAI chatbots use to loneliness, GCU → MI → NE → LL (Ind3; H5) versus GCU → UT → NE → LL (Ind4; H6), whose difference did not reach statistical significance (Δ = 0.012, p = 0.085, 95% CI [−0.001, 0.027]). Therefore, the evidence does not support a differential impact between the two dual-chain mechanisms at the full mediation level; rather, the results indicate that the two pathways may operate in parallel with comparable overall strength. Taken together, these findings imply that the psychosocial correlates of GenAI chatbots use are not unidimensional, but reflect an interplay of cognitive and affective processes that co-occur in shaping loneliness-related outcomes.

Additionally, the R^2^ value for Loneliness in this study was 0.035, indicating that the model explains only a limited variance of loneliness. The findings statistically support the existence of two parallel chained mediation mechanisms, but do not suggest strong predictive power or high explanatory force for loneliness. Given that loneliness is typically determined by multiple factors including social support, quality of offline interactions, personality, and mental health, this study focused solely on the two psychological process chains: “misinformation exposure/trust—negative emotions”. Consequently, the low explanatory power represents a limitation that must be acknowledged. Future research should incorporate more comprehensive key antecedent variables and contextual factors to examine the boundary conditions and incremental explanatory power of this mechanism within a more complete model.

Collectively, this study proposes a dual-path cognitive–affective framework linking GenAI chatbots use to loneliness via perceived misinformation exposure and user trust, both operating through negative emotions, thereby extending the S-O-R framework to AI–human interaction. However, the model explains a limited proportion of variance in loneliness (R^2^ = 0.035), and a competing model including a direct GCU→LL path shows no additional explanatory gain (β = 0.006, p = 0.900; R^2^ unchanged). Thus, the findings should be interpreted as statistically supported but small indirect associations that are most informative for mechanism identification rather than strong prediction. In relation to the emerging public health debate on “AI psychosis,” our results suggest that perceived cognitive uncertainty during GenAI interactions, particularly exposure to misleading or difficult to verify outputs—may serve as a risk signal worth monitoring.

### Implications

From the public health perspective, these findings offer significant insights. First, as GenAI becomes more prevalent, the psychological burdens users experience during daily use, such as loneliness, have transcended mere technological adaptation issues and evolved into potential health risks. This aligns with recent warnings and concerns about “AI psychosis”, which suggests that AI interactions are associated with cognitive confusion, emotional distress, and even psychiatric symptoms. Second, the results suggest public health interventions must balance information discernment with emotional regulation: on one hand, digital literacy education should enhance users’ critical evaluation skills to prevent cognitive harm from misinformation; on the other, social support and emotional counseling should be strengthened to mitigate loneliness and negative emotions stemming from over-reliance on AI. This dual-dimensional approach offers a more comprehensive response to mental health challenges in the AI era. Practically, a harm-reduction approach may help mitigate loneliness-related risks among frequent GenAI users. On the information side, platforms could implement uncertainty signaling, source attribution, and verification prompts for ambiguous or potentially misleading outputs. On the psychosocial side, interventions may include balanced-use features, encouragement of offline social contact when distressed, and mental-health referral guidance when appropriate.

### Limitations

This study has several limitations. First, although the hypothesized paths are statistically supported, the model explains a limited proportion of variance in loneliness (R^2^ = 0.035). Therefore, the practical significance of the observed effects should be interpreted cautiously, and the framework is better suited for mechanism identification than strong prediction of loneliness. Second, cross-sectional self-report designs preclude causal inference and temporal sequencing; all findings can only be interpreted as associations and statistical indirect effects. Third, the sample was recruited via WenJuanXing using an online, non-probability convenience approach, and the findings should be interpreted as most relevant to active GenAI users in China with similar characteristics and should not be generalized to the broader population or internationally. Finally, the absence of longitudinal tracking data and cross-cultural comparisons limits conclusions about temporal dynamics and cross-cultural generalizability.
